# Natural ageing primarily affects the initial response to a sustained walking perturbation but not the ability to adapt over time

**DOI:** 10.3389/fphys.2023.1065974

**Published:** 2023-02-23

**Authors:** S. B. Swart, A. R. den Otter, C. J. C. Lamoth

**Affiliations:** Department of Human Movement Sciences, University of Groningen, University Medical Centre Groningen, Groningen, Netherlands

**Keywords:** split-belt adaptation, flexibility, adaptability, lifespan, singular spectrum analysis

## Abstract

The ability to flexibly respond and adapt the walking pattern over time to unexpected gait perturbations is pivotal for safe and efficient locomotion. However, these abilities might be affected by age due to age-related changes in sensorimotor functioning. In this cross-sectional lifespan study, we used a split-belt paradigm to determine how age affects the initial response (i.e., *flexibility*)—and the ability to adapt after prolonged exposure—to a sustained gait perturbation. Healthy adults (N = 75) of different ages (12–13 per decade) were included and walked on a split-belt treadmill, in which a sustained gait perturbation was imposed by increasing one of the belt speeds. Linear regression models, with the evoked spatiotemporal gait asymmetry during the early perturbation and late adaptation, were performed to determine the effects of age on the flexibility and adaptability to split-belt walking. Results showed that the flexibility to respond to an unexpected perturbation decreased across the lifespan, as evidenced by a greater step length asymmetry (SLA) during the early perturbation phase. Despite this reduced flexibility in step lengths, late adaptation levels in SLA were comparable across different ages. With increasing age, however, subjects needed more steps to reach a stable level in SLA. Finally, when the belts were set to symmetrical speeds again, the magnitude of SLA (i.e., *the aftereffects*) increased with age. Collectively, these findings suggest that natural ageing comes with a decrease in gait flexibility, while the ability to adapt to split-belt walking was not affected by age—only how adaptation was achieved.

## 1 Introduction

Healthy humans can control their walking adaptively to ensure that constant task performance is maintained in the light of continuously changing demands—allowing us to walk, e.g., on new shoes or irregular terrains, without much difficulty. Locomotor adaptability and flexibility may nonetheless change throughout the adult lifespan due to age-related changes in the sensory and neuro-motor systems (Callisaya et al., 2009), such as progressive reductions in muscle mass and motor unit remodelling (Charlier et al, 2015; Piasecki et al., 2016). While these age-related declines in motor functioning can have a relatively early onset (i.e., *40–50 yrs*) and contribute to changes in the walking pattern at an older age, such as slower gait speed, reduced step lengths, and increased double support times (Callisaya et al., 2009), it is not fully understood how natural ageing affects locomotor flexibility and adaptability. To better understand *if* these age-related changes affect locomotor control, it is imperative to map locomotor flexibility and adaptability across different ages.

Locomotor flexibility and adaptability are essential in daily life locomotion. *Flexibility* reflects the ability to generate a coordinated (loco)motor solution immediately after the initial exposure to a new task (Ranganathan et al., 2020). While this initial response is essential to maintain a functional task performance, further optimization and fine-tuning need to be achieved through *adaptability*, i.e., the ability to generate a more structural and coordinated (loco)motor solution to the new task after sustained exposure (Krakauer et al., 2019). Together, locomotor flexibility and adaptability make transitions between different task contexts possible without much difficulty during daily life locomotion, which in turn optimizes the locomotor task performance.

The split-belt treadmill paradigm allows researchers to assess locomotor flexibility and adaptability by setting the two independent belts to asymmetrical speeds (Reisman et al., 2005). Subjects initially respond to these new task demands by walking asymmetrically but re-establish symmetry in step lengths over time. Minimizing the initial asymmetry and re-establishing symmetry over time are important since gait asymmetry increases the metabolic and mechanical demands of walking (Finley et al., 2013; Selgrade et al., 2017)—an inability to do so is therefore considered signs of reduced locomotor flexibility and adaptability, respectively. Optimizing the task performance thus requires the flexibility to shift between task contexts and the adaptability to recalibrate the normal walking pattern. The initial level of step length asymmetry that is observed immediately following exposure to the split-belt condition is reflective of the flexibility to switch between task contexts. The number of steps (i.e., *repetitions*) needed to reach a stable level in gait asymmetry gives insights into how gait adaptations were achieved throughout the split-belt phase. When the belts are set to symmetrical speeds again, step lengths show robust—whereas stance times show minor—asymmetry in the opposite direction of adaptation (i.e., *the aftereffects*) (Reisman et al., 2005), indicating the extent to which spatial gait parameters were recalibrated during split-belt adaptation (Torres-Oviedo et al., 2011).

Over the past decade, studies have probed the effects of older age on locomotor adaptation comparing distinct groups of young and older adults (Bruijn et al., 2012; Roemmich et al., 2014; Malone and Bastian, 2016; Sombric et al., 2017; Vervoort et al., 2019). However, we recently showed in a cross-sectional lifespan study that changes in the control of temporal step characteristics during split-belt adaptation already occur around the age of 40 (Vervoort et al., 2020). Using the margin of stability concept, such age-related changes in temporal step characteristics were with associated increased margins in mediolateral dynamic stability (Vervoort et al., 2020). In the current study, we used the same data set (Vervoort et al., 2020) but now assess the asymmetry in spatiotemporal parameters to determine how age affects **(i)** the ability to respond adequately to a sudden gait perturbation (i.e., *flexibility*) and **(ii)** the ability to adapt after prolonged exposure to this perturbation (i.e., *adaptability*). In addition, the number of steps needed to reach a stable level of gait asymmetry (i.e., *the number of steps to plateau*), and the size of the aftereffects following perturbation removal, were assessed to gain insights into how adaptation was achieved. Singular Spectrum Analysis (SSA) was used to estimate the adaptation trends (Swart et al., 2022). This non-parametric approach can achieve accurate fits for the idiosyncratic time series obtained from individual subjects and allows us to map changes in locomotor flexibility and adaptability across the adult lifespan.

## 2 Methods

### 2.1 Participants

The dataset of this cross-sectional study has been previously used to probe adaptive control of dynamic balance across the adult lifespan (Vervoort et al., 2020). This dataset includes a sample (*N* = 75, age range: 18–79) of 12–13 healthy adults per decade (see supplement (Vervoort et al., 2020) for participant characteristics). The inclusion criterion was being able to walk without walking aids. Exclusion criteria were previous experience with split-belt walking, recent orthopaedic surgery (<2 years), neurological disorders, (neuro) psychiatric disorders, and medication that might affect walking. Experimental procedures were approved by the local Ethical Committee (Department of Human Movement Sciences, Groningen) and were per the principles outlined in the declaration of Helsinki (World Medical Association, 2013). All participants provided written informed consent before experimentation.

### 2.2 Instrumentation

**FIGURE 1:**
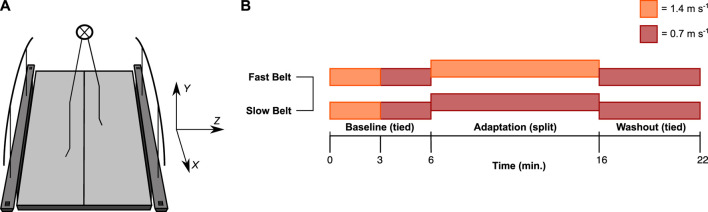
Experimental setup. **(A)** Participants walked on a split-belt treadmill. The data was analysed in an *XYZ* coordinate system, with the *x*-axis in the fore-aft, *z*-axis in the medial-lateral, and *y*-axis in the vertical direction (Wu & Cavanagh, 1995). **(B)** The baseline consisted of two 3-min bouts of fast (1.4 m s^−1^) and slow (0.7 m s^−1^) tied-belt walking. The fast and the slow speed were randomly assigned to either the left or right leg during the adaptation phase. During the washout phase, the speed of both belts was 0.7 m s^−1^ again.

Participants walked on an instrumented split-belt treadmill (M-Gait, Motek force Link, Amsterdam, NL) (Figure 1A). 3D ground reaction forces (GRF) (N) and moments of force (Nm) were measured by 2-individual force plates embedded in each treadmill belt and recorded with D-Flow software (Motek force Link, Amsterdam, NL) at a sampling frequency of 1,000 Hz.

### 2.3 Experimental procedures and protocol

Participants were secured in a harness that did not provide bodyweight support during walking. The protocol started with two 3-min bouts of tied-belt walking at 1.4 and 0.7 m s^−1^, respectively (Figure 1B). Next, participants walked in the split-belt configuration for 10 min. The fast belt at 1.4 m s^−1^ and the slow belt at 0.7 m s^−1^ for each participant was randomly assigned to the left or right leg—henceforth referred to as the fast and slow leg. After the adaptation phase, participants walked for an additional 6 min in a tied-belt configuration at a velocity of 0.7 m s^−1^ (Figure 1B). Participants were instructed to look straight ahead and not to use the handrails as this affects locomotor adaptation (Buurke et al., 2019).

### 2.4 Data analysis

Custom-made MATLAB (version r2016b; The Mathworks Inc., Natick, MA) routines were used for the data analysis. All data were analyzed in an *XYZ* coordinate system, with the *x*-axis in the fore-aft, *z*-axis in the medial-lateral, and *y*-axis in the vertical direction (Figure 1A) (Wu and Cavanagh, 1995). The force plate data were filtered with a 2nd order low-pass Butterworth filter (cf. = 15 Hz). Initial foot contacts and toe-offs were identified by finding the moment when the vertical GRF of an individual force plate crossed the detection threshold of 50 N. Unfiltered moments of force in the *x* and *z* direction were divided by the unfiltered vertical GRF to calculate *z* and *x* center of pressure (COP) positions (m). The COP data of the individual belts were combined to ensure continuous monitoring by scaling the COP position to the magnitude of its vertical GRF (Buurke et al., 2018).

The combined COP position was used to compute the step lengths (m), which were defined as the difference between the COP*x* position of the fast (SLF) and slow (SLS) leg at initial foot contact. Step lengths were divided by leg length (m) for normalization (Hof, 1996). Swing times (% total stride duration) were defined for the fast (STF) and slow (STS) leg as the time between ipsilateral toe-off and foot strike, expressed as a percentage of total stride duration. Lastly, asymmetry ratios were computed for the step lengths and swing times using Eq. 1 (Reisman et al., 2005):

$$Asymmetry\left(i\right)=\frac{Fast\left(i\right)-Slow\left(i\right)}{Fast\left(i\right)+Slow\left(i\right)}$$ (1)

Since subjects can use different strategies to achieve similar asymmetry values (e.g., *fictitious normalized step length values of* ***0.6*** *and* ***0.4*** *yield the same asymmetry as* ***0.3*** *and* ***0.2***), both the asymmetry ratios and the spatiotemporal parameters of individual legs were included in the formal analysis. The former were used to characterize the task-goal performance during split-belt adaptation, while the latter were used to characterize how changes in asymmetry were achieved.

### 2.5 Singular Spectrum Analysis (SSA)

**FIGURE 2:**
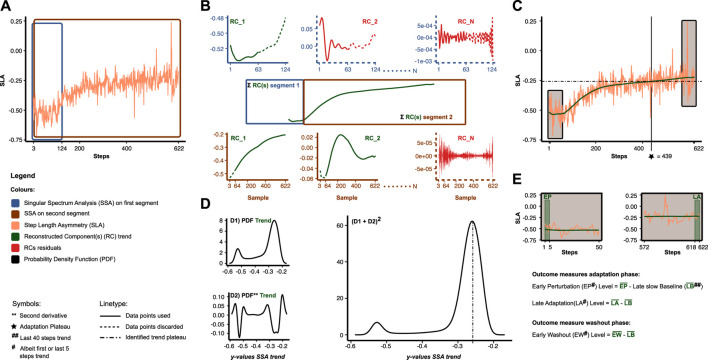
Overview of data analysis and outcome measures. **(A)** Empirical time series of step length asymmetry (SLA) (orange). The Singular Spectrum Analysis (SSA) is applied separately to two overlapping segments (blue and brown). **(B)** For the SSA in each segment, we reconstructed components (RC). Trend RC(s) (green) are characterized by a high contribution in the low-frequency domain, while residual RCs (red) are characterized by a relatively lower contribution in the low-frequency domain. The trend RC(s) per segment are summed and merged to acquire the entire trend. **(C)** Final result of the SSA. **(D)** To assess the adaptation plateau, we first estimate the probability density function (PDF) of the SSA trend (D1). Subsequently, we add the second derivative of the PDF (D2) and square the result (D1+D2)^2^. Peaks in the latter are used to identify plateau(s) (point-dashed lines) in the SSA trend. Adaptation plateau (star) was defined as the crossing point between SSA trend (green) and plateau. **(E)** Other outcome measures. Levels were computed by averaging albeit the first or last 5 data points of the SSA trend relative to late slow baseline.

For the aims of this study, we were interested in quantifying the adaptation time course, early perturbation level, late adaptation level, and early washout level during and after split-belt walking. First, adaptation trends were identified from the raw time series with the non-parametric SSA to this end. In addition, trends for the baseline phases were identified to normalize the early perturbation, late adaptation, and early washout levels for baseline step lengths and swing times. Figure 2 illustrates the general concepts of the SSA procedure. The SSA is suitable for adaptation time series to separate the trend from unwanted noise and other potentially high-frequency components with unknown period (Alexandrov, 2009; Swart et al., 2022). The SSA starts with the so-called *embedding* step, where we construct a Hankel/Trajectory matrix by sliding a window ⌊N/2⌋ with a lag of 1 across the time series *Y*_*N*_ (e.g., *a raw SLA time series*). Each column in the Hankel matrix corresponds to the part of *Y*_*N*_ in the sliding window. The next step is to *decompose* the Hankel matrix via an eigendecomposition. That provides us with orthogonal matrices of (transposed) eigenvectors and a diagonal matrix of eigenvalues in decreasing norm. The orthogonal matrix of eigenvectors is used to obtain principal component vectors (PV). The next step is to *identify* the PV(s) associated with the trend. Via spectral analysis, we select PV(s) with a high contribution in the low-frequency domain for the trend in a data-driven manner. Lastly, we *reconstruct* the trend with the PV(s) identified as slow-varying. Figure 2B illustrates several reconstructed components (RC) of PVs, summing the RCs of the PV(s) identified as trend (green RCs in Figure 2B) yield the trend of the time series (Figure 2C). This standard SSA procedure was applied to two overlapping segments of the original time series for the adaptation and washout phases (Figure 2A), to improve the reconstruction of the initial steep part of adaptation and washout phases (Swart et al., 2022). A brief mathematical description of how the SSA works is provided in the supplementary—we refer to a recent paper on this topic (Swart et al., 2022) for a more detailed description.

### 2.6 Outcome measures

Figures 2C, D illustrates how we quantified the adaptation time course of the step length and swing time asymmetry (STA) trends. In the adaptation phase, participants eventually reach a plateau in gait asymmetry as little or no adaptation occurs thereafter. Previous studies have averaged the asymmetry time series in predefined bins and compared these bins with the baseline to assess the number of steps people needed to reach the plateau (Bruijn et al., 2012; Malone and Bastian, 2016). While this provides information on how people adapt over rough periods of time, the size of the bin could bias it. To quantify the plateau(s), we, therefore, adapted a non-parametric method originally used in biological data sets (Tyson et al., 2015). *First*, we used kernel density estimation to estimate the probability density function (PDF) of the SSA trend. Peaks in this PDF represent a high probability that *y*-coordinate values in the SSA trend reside at that value (i.e., *a potential plateau*). *Secondly*, we computed the second derivative of the PDF via finite differences (i.e., *the discrete Laplacian*) and added this back to the original PDF to amplify portions of the trend at which there are large changes in direction. In addition, we squared the results to make all values positive. *Thirdly*, we identified the local minima and maxima in the squared signal, i.e., local minima are preceded and succeeded by a higher value, and conversely, local maxima are preceded and succeeded by a lower value. *Fourthly*, we considered a peak in the squared signal to be a plateau when the difference between the minimum preceding the maximum and the difference between the minimum succeeding said maximum was larger than the standard deviation of the squared signal. The steps to plateau were now defined as the step at which the SSA trend and plateau crossed—a detailed description identifying this crossing point is provided in the supplementary. The number of steps to the plateau was divided by the cadence (steps min. ^-1^) up until the plateau was reached to calculate the adaptation time (min.).

The trends from SLA, SLF, SLS, STA, STF, and STS were used to compute the early perturbation, late adaptation, and early washout levels. Higher levels of spatial asymmetry during the early perturbation and late adaptation levels were considered signs of reduced locomotor flexibility and adaptability, respectively. Early perturbation, late adaptation, and early washout levels were defined as the trend average of the first/last 5-adaptation/washout steps minus the trend average of the last 40 slow baseline steps (Sombric and Torres-Oviedo, 2020).

### 2.7 Statistical analysis

Statistical analysis was performed in R Studio OS version 1.2.5033 (RStudio, Boston, MA, United States) with R version 3.6.1 (R-Project, Vienna, AT). Linear regression models were used with age as an independent variable and the steps to adaptation plateau, adaptation time, early perturbation, late adaptation, and early washout levels of each spatiotemporal parameter as dependent variables. Residuals of each model were visually checked for violations of homoscedasticity. Multiple testing was corrected using the Benjamini–Hochberg procedure with a false discovery rate of 0.05 (Benjamini and Hochberg, 1995).

## 3 Results

### 3.1 The effects of age on locomotor flexibility and adaptability during split-belt walking

**FIGURE 3:**
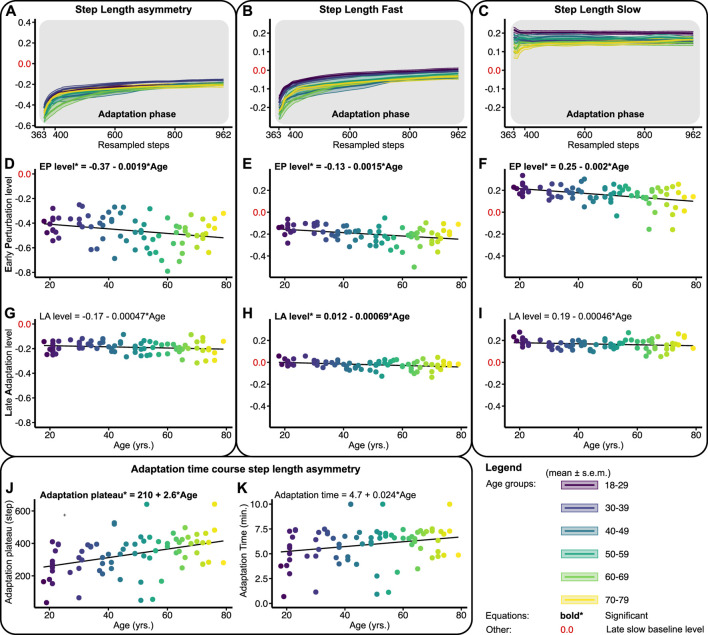
Baseline adjusted outcome measures step length asymmetry (SLA) and step lengths (SLF/SLS) during the adaptation phase. Significant relations are in bold and red values on *y*-axes signify the late slow baseline level. NB *y*-range of all the level outcomes in Figures 3, 5 are equal for SLF and SLS and equal for SLA. **(A–C)** Group-averaged adaptation trends for SLA, SLF, and SLS, shaded areas around the mean represent the s.e.m. Adaptation trends were resampled to 600 data points for visualization only **(D–F)** Early perturbation (EP) levels for SLA, SLF, and SLS. **(G–I)** Late adaptation (LA) levels for SLA, SLF, and SLS. **(J, K)** Shows the time course of SLA. Adaptation plateau, i.e., the step at which the SLA plateaus, and adaptation times (min.), i.e., the adaptation plateau divided by the cadence.

Figure 3 shows the adaptation trends, early perturbation, and late adaptation levels for SLA, SLF, and SLS relative to the slow late baseline—outcomes of the linear regression models are provided in Table 1. To probe locomotor flexibility, we assessed early perturbation levels in SLA, SLF, and SLS. No severe violations of homoscedasticity for the linear regression models were found. Upon split-belt exposure subjects walked with a negative SLA by decreasing the step length on the fast and increasing the step length on the slow belt compared to late slow baseline (Figures 3A–C). The early perturbation level of SLA decreased significantly across the lifespan with 0.0019 per year (Figure 3D; Table 1). Early perturbation levels for SLF and SLS decreased significantly across the lifespan as well with 0.0015 and 0.002, respectively (Figures 3E, F; Table 1). With advancing age, subjects thus decreased their step length on the fast belt more and increased their step length on the slow belt less, resulting in a larger SLA during early perturbation (i.e., *a reduced flexibility*).

**TABLE 1:** Results of the linear regression models for step length asymmetry (SLA), step length fast (SLF), step length slow (SLS), swing time asymmetry (STA), swing time fast (STF), and swing time slow (STS) with age as the independent variable. Significant *p*-values are in bold.

Outcome measure	Parameter	Intercept (standard error)	t-value intercept (*p*-value)	Beta coefficient (standard error)	t-value beta	R2	*p*-value
Adaptation plateau (step)							
	SLA	210 (37)	5.56 (<0.001)	2.6 (0.72)	3.64	0.15	**0.00051**
	STA	220 (48)	4.56 (<0.001)	2.4 (0.93)	2.63	0.087	**0.01**
Adaptation time (min.)							
	SLA	4.7 (0.63)	7.56 (<0.001)	0.024 (0.012)	1.99	0.051	0.051
	STA	5 (0.84)	5.95 (<0.001)	0.021 (0.016)	1.27	0.022	0.21
Early perturbation level							
	SLA	−0.37 (0.038)	−9.80 (<0.001)	−0.0019 (0.00074)	−2.51	0.08	**0.014**
	SLF	−0.13 (0.025)	−5.06 (<0.001)	−0.0015 (0.00048)	−3.15	0.12	**0.0024**
	SLS	0.25 (0.027)	9.40 (<0.001)	−0.002 (0.00053)	−3.70	0.16	**0.00042**
	STA	0.24 (0.020)	12.19 (<0.001)	−0.0016 (0.00039)	−4.01	0.18	**0.00015**
	STF (%)	11 (0.98)	11.54 (<0.001)	−0.055 (0.019)	−2.88	0.1	**0.0052**
	STS (%)	−7.1 (0.94)	−7.61 (<0.001)	0.06 (0.018)	3.31	0.13	**0.0015**
Late adaptation level							
	SLA	−0.17 (0.016)	−10.28 (<0.001)	−0.00047 (0.00032)	−1.50	0.03	0.14
	SLF	0.012 (0.012)	0.98 (0.33)	−0.00069 (0.00024)	−2.87	0.1	**0.0054**
	SLS	0.19 (0.015)	12.47 (<0.001)	−0.00046 (0.00029)	−1.57	0.033	0.12
	STA	0.19 (0.016)	11.68 (<0.001)	−0.00085 (0.00031)	−2.71	0.091	**0.0084**
	STF (%)	10 (0.83)	12.51 (<0.001)	−0.051 (0.016)	−3.13	0.12	**0.0025**
	STS (%)	−4.1 (0.56)	−7.23 (<0.001)	0.014 (0.011)	1.31	0.023	0.2
Early washout level							
	SLA	0.15 (0.042)	3.55 (<0.001)	0.0021 (0.00083)	2.51	0.079	**0.014**
	SLF	0.067 (0.015)	4.50 (<0.001)	−0.00076 (0.00029)	−2.63	0.086	**0.01**
	SLS	−0.032 (0.026)	−1.23 (0.22)	−0.002 (0.00051)	−3.19	0.17	**0.0002**
	STA	−0.062 (0.035)	−1.78 (0.079)	0.0017 (0.00067)	2.57	0.083	**0.012**
	STF (%)	−2.6 (1.5)	−1.70 (0.093)	0.066 (0.029)	2.23	0.064	0.029**
	STS (%)	2 (1.3)	1.56 (0.12)	−0.062 (0.025)	−2.46	0.076	**0.016**

^a^ Critical values were computed with (i/m)*Q, where i was the rank, m the number of tests (22), and Q the false discovery rate (0.05). **Significant alpha level following the Benjamini–Hochberg procedure.

Throughout the adaptation phase, subjects began to walk more symmetrically by increasing the step length on the fast belt compared to early perturbation (Figures 3A–C). To probe locomotor adaptability, late adaptation levels in SLA, SLF, and SLS were assessed. The late adaptation level of SLA did not significantly change across the lifespan (Figure 3G; Table 1). Although significant for SLF, late adaptation levels for SLF and SLS showed marginal (i.e., *0.00069 and 0.00046, respectively*) changes per year across the lifespan (Figures 3H, I; Table 1). Subjects thus achieved similar levels in SLA, SLF, and SLS at the end of split-belt adaptation, irrespective of age. This illustrates that regardless of age, people were able to adapt to the same level, implying that adaptability is not affected by age. The time course of SLA adaptation, however, did change with age. The number of steps subjects needed to reach a plateau in SLA increased significantly across the lifespan by 2.6 steps per year (Figure 3J; Table 1). Yet, the actual time it took subjects to reach said plateau did not significantly change across the lifespan (Figure 3K; Table 1). These findings illustrate that age does not affect the ability to adapt step lengths over time, only how the adaptations were achieved.

**FIGURE 4:**
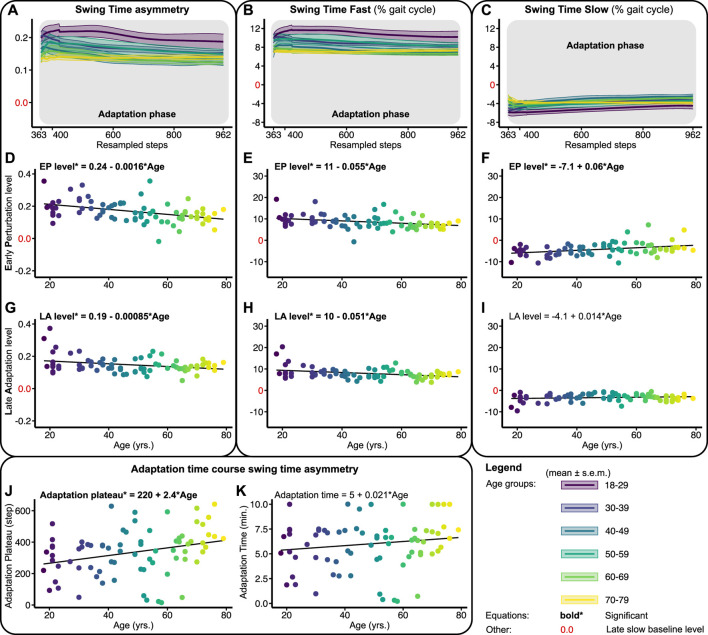
Baseline adjusted outcome measures swing time asymmetry (STA) and swing times (STF/STS) during the adaptation phase. Significant relations are in bold and red values on *y*-axes signify the late slow baseline level. NB *y*-range of all the level outcomes in Figures 4, 6 are equal for STF and STS and equal for STA. **(A–C)** Group-averaged adaptation trends for STA, STF, and STS, shaded areas around the mean represent the s.e.m. Adaptation trends were resampled to 600 data points for visualization only **(D–F)** Early perturbation (EP) levels for STA, STF, and STS. **(G–I)** Late adaptation (LA) levels for STA, STF, and STS. **(J, K)** Shows the time course of STA. Adaptation plateau, i.e., the step at which the STA plateaus, and adaptation times (min.), i.e., the adaptation plateau divided by the cadence.

Figure 4 shows the adaptation trends, early perturbation, and late adaptation levels for STA, STF, and STS relative to the slow late baseline. Upon split-belt exposure, subjects walked with a positive STA by increasing the swing time on the fast and decreasing the swing time on the slow belt compared to the late slow baseline (Figures 4A–C). The early perturbation level of STA decreased significantly across the lifespan with 0.0016 per year (Figure 4D; Table 1). For the individual legs, the early perturbation level of STF decreased significantly with 0.055% per year (Figure 4E; Table 1), while the early perturbation level of STS increased significantly across the lifespan by 0.06% per year (Figure 4F; Table 1). With advancing age, subjects thus increased their swing time on the fast belt less and decreased their swing time on the slow belt less, resulting in a smaller STA during early perturbation.

Throughout the adaptation phase, subjects began to walk slightly more symmetrical by decreasing the swing time on the fast and increasing the swing time on the slow belt compared to early perturbation (Figures 4A–C). Late adaptation levels for STA and STF decreased slightly but significantly across the lifespan (Figures 4G, H; Table 1). Late adaptation level of STS did not significantly change across the lifespan (Figure 4I; Table 1). With advancing age, subjects thus walked with slightly more symmetrical swing times at the end of adaptation. In addition, the time course of STA adaptations differed across the lifespan as well. The number of steps subjects needed to reach a plateau in STA increased significantly across the lifespan by 2.4 steps per year (Figure 4J; Table 1). The time subjects needed to reach the plateau in STA did, however, not significantly change across the lifespan (Figure 4K; Table 1). The ability to adapt swing times over time was thus not affected by age, but subjects needed more steps to reach a plateau in STA with advancing age.

### 3.2 The effects of age on locomotor aftereffects during the washout phase

**FIGURE 5:**
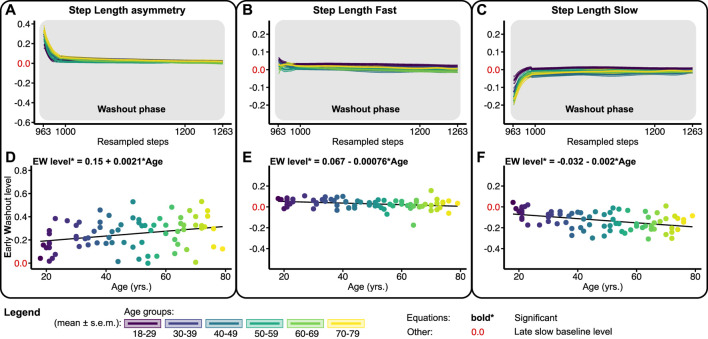
Baseline adjusted outcome measure for step length asymmetry (SLA) and step lengths (SLF/SLS) during the washout phase. Significant relations are in bold and red values on *y*-axes signify the late slow baseline level. NB *y*-range of all the level outcomes in Figures 3, 5 are equal for SLF and SLS and equal for SLA. **(A–C)** Group-averaged washout trends for SLA, SLF, and SLS, shaded areas around the mean represent the s.e.m. Washout trends were resampled to 300 data points for visualization only **(D–F)** Early washout (EW) levels for SLA, SLF, and SLS.

Figure 5 shows the washout trends and early washout levels for SLA, SLF, and SLS relative to the slow late baseline. At the beginning of the washout phase, subjects walked with a positive SLA by slightly increasing the step length on the fast and decreasing the step length on the slow belt compared to late slow baseline (Figures 5A–C). The early washout level of SLA increased significantly across the lifespan with 0.0021 per year (Figure 5D; Table 1). Early washout levels for SLF and SLS decreased significantly across the lifespan with 0.00076 and 0.002, respectively, per year (Figures 5E, F; Table 1). These findings show that the magnitude of spatial aftereffects increases with age.

**FIGURE 6:**
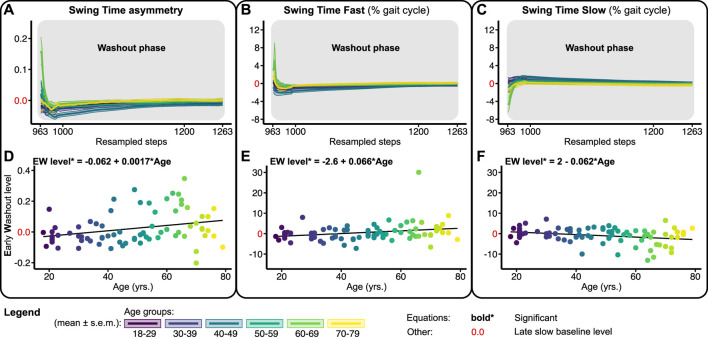
Baseline adjusted outcome measure for swing time asymmetry (STA) and swing times (STF/STS) during the washout phase. Significant relations are in bold and red values on *y*-axes signify the late slow baseline level. NB *y*-range of all the level outcomes in Figures 4, 6 are equal for STF and STS and equal for STA. **(A–C)** Group-averaged washout trends for STA, STF, and STS, shaded areas around the mean represent the s.e.m. Washout trends were resampled to 300 data points for visualization only **(D–F)** Early washout (EW) levels for STA, STF, and STS.

Figure 6 shows the washout trends and early washout levels for STA, STF, and STS relative to the slow late baseline. At the beginning of the washout phase, most subjects walked with a small negative STA (Figure 6A). This early washout level increased significantly across the lifespan with 0.0017 per year (Figure 6A; Table 1). As such, early washout levels of STA were more often positive for the older participants. Early washout levels for STF increased significantly with 0.066% (Figure 6E; Table 1), while early washout levels of STS decreased significantly across the lifespan with 0.062% per year (Figure 6F; Table 1). Overall, these results suggest that older adults show little adjustments in swing times during split-belt walking. In fact, some of the older participants do not recalibrate their swing times at all as evidenced by positive STA values during the early washout. In contrast, younger adults make larger adjustments in their swing times (Figure 4), resulting in small aftereffects upon return to tied-belt walking.

## 4 Discussion

In this cross-sectional study, we assessed how natural ageing affects locomotor flexibility and adaptability during split-belt walking. Walking in the split-belt configuration necessitates both *flexibility* to adjust the gait at the early perturbation and *adaptability* to make structural gait adaptations over time. The flexibility to adjust the step lengths decreased with age, as indicated by a larger SLA during early perturbation. This suggest that with advancing age, healthy adults have greater difficulties transitioning from tied-to-split belt walking. Irrespective of age, final performance of all subjects was comparable at the end of adaptation, but older adults needed more steps to reach the same level of symmetry. When subjects returned to tied-belt walking, the magnitude of spatial aftereffects increased with age, implying that older adults recalibrated their step lengths more during split-belt adaptation. Overall, these findings suggest that advancing age primarily comes with reduced locomotor flexibility, while locomotor adaptability is preserved. In other words, it takes more steps to reach a stable adapted state again after a perturbation with advancing age.

The reduced locomotor flexibility in step length modulation with advancing age appears unrelated to an inability to walk more symmetrical. Early perturbation levels showed an age-related increase in SLA following the switch from tied-belt to–split-belt walking, while late adaptation levels revealed that SLA did not considerately differ across ages at the end of adaptation. Conjointly these findings underscore that age primarily affects the ability to transition between different locomotor tasks flexibly. Previous studies have shown that the SLA during the early perturbation is the combined result of shortening the step on the fast and lengthening the step on the slow belt compared to baseline walking (Reisman et al., 2005). Results showed that step lengths on the fast belt decreased more, while step lengths on the slow belt increased less with advancing age. With increasing age, the coordinative possibilities to transition from tied-to-split belt walking thus appear to shift toward step shortening, while the possibilities to step lengthening are less utilized or cannot be utilized instantaneously. Such step-shortening strategies commonly occur in older adults as a way to safeguard dynamic balance (Winter et al, 1990). Interestingly, recent work showed that the ability to make quick step adjustments was more impaired in fallers than in non-fallers (Caetano et al., 2018; Ekvall Hansson et al., 2021). Therefore, reduced locomotor flexibility in step length modulation with advancing age could be a precursor for fall events in daily life.

In addition to age-related changes in locomotor capacity, changes in attentional capacity could also contribute to the reduced spatial flexibility that was observed. Switching from tied-to-split belt walking can be considered cognitively demanding as the initial epoch of split-belt adaptation is adversely affected by distraction, suggesting the involvement of attentional mechanisms when shifting between locomotor tasks (Malone and Bastian, 2010). In line with this, recent work also showed that the performance on a cognitive task (i.e., *the auditory Stroop task*) worsens with advancing age during the early exposure to split-belt adaptation, whereas the initial level of SLA was not affected by age (Vervoort et al., 2019). This prioritization of the motor task suggests that the shift from tied-to-split belt walking requires a higher attentional load with advancing age. In addition, it has been shown that attentional capacity decreases linearly across the adult lifespan (McAvinue et al., 2012). Arguably, these findings suggest that age-related changes in attentional capacity could also hamper the ability to transition flexibly between tasks.

Following sustained exposure, step lengths became more symmetrical, and final performance after 10 min of split-belt walking was comparable across ages. This finding is consistent with previous studies (Roemmich et al., 2014; Vervoort et al., 2019) and shows that people can reduce their SLA in response to sustained split-belt exposure irrespective of age. However, age did affect how adaptation was achieved. Results showed that the number of steps subjects needed to reach a plateau in SLA increased significantly by 2.6 steps per year, indicating that more task repetitions were needed with advancing age to complete adaptation. Since the older adults in this cohort walked at a higher cadence (Vervoort et al., 2020), the time it took them to reach the plateau in SLA was comparable to younger adults. Although previous studies stated that older adults (M±s.d: 73.1 ± 4.7 (Bruijn et al., 2012), 77.2 ± 2.8 (Sombric et al., 2017)) adapt their step lengths slower towards symmetry, our results suggest that healthy older adults do not necessarily adapt their step lengths *slower* but need more step repetitions within the new environment to reach the same level of performance as young adults.

When people return to tied-belt walking during the washout phase, SLA aftereffects occur in the opposite direction of adaptation (Reisman et al., 2005), indicating that an existing motor pattern was recalibrated during the adaptation phase (Krakauer et al., 2019). Previous work has shown that larger initial errors and the total adaptation magnitude (i.e., *early perturbation minus late adaptation level*) are related to larger aftereffects in SLA (Torres-Oviedo and Bastian, 2012; Wood et al., 2020). Indeed, following larger SLA during early exposure, and comparable SLA during late adaptation, the magnitude of SLA aftereffects increased with age. This suggests that healthy older adults still have adaptive space they can utilize to attain a similar final level of performance as younger adults, even though the initial asymmetry they had to overcome was larger than younger adults. Next to the sensorimotor recalibration of upper extremity motor behaviour (Vandevoorde and Orban de Xivry., 2019), the larger SLA aftereffects with age illustrate that sensorimotor recalibration of locomotor behaviour is also unaffected by age. In fact, the larger aftereffects imply that a more profound recalibration was required to arrive at similar adaptive states as the transition between different task contexts became more difficult with advancing age.

As opposed to SLA, adaptation did not occur in swing times as aftereffects were albeit small or in the same direction of adaptation. A previous study found that asymmetry in stance times increases during early perturbation for greater belt-speed differences, whereas the ensuing aftereffects remain marginal and consistent across those conditions (Yokoyama et al., 2018), which illustrates that increases in STA during the adaptation phase scale linearly with task difficulty. While one may expect that the task was more difficult for older adults, results showed that the youngest adults had a consistently higher STA throughout the adaptation phase. The fact that older adults walked at a higher cadence (Vervoort et al., 2020), with a reduced ability to modulate step lengths, may have limited them to increase their swing times to a similar extent as younger adults (see Figure 4B). Previous studies have shown that younger adults primarily adjust their swing times, while older adults show larger changes in the swing speed instead (Bruijn et al., 2012; Vervoort et al., 2019). Since the present results show that the shift towards decreased STA occurs markedly after age 30 (Figures 4A–C), the underlying mechanism of the different temporal strategy at higher age could be associated with age-related changes in the sensory and neuromotor systems occurring around that age.

While several studies assessed the effects of older age on locomotor adaptation, we expand those findings here by assessing both locomotor flexibility and adaptability across different ages. Changes in locomotor flexibility and adaptability followed linear-like tendencies with increasing heterogeneity across the adult age continuum (i.e., *18–79 yrs*)*.* Such age-related changes were primarily evident in the flexibility required immediately following the switch between locomotor tasks, while the adaptability over time remained largely unaffected by age. Overall this cross-sectional study showed that the switch between locomotor tasks was more detrimental to task performance with increasing age, necessitating a more profound recalibration of the walking pattern to arrive at similar adapted states.

## Data Availability

The original contributions presented in the study are included in the article/Supplementary materials, further inquiries can be directed to the corresponding author.

## References

[B1] Alexandrov T. A. (2009). Method of trend extraction using singular spectrum analysis. Revstat Stat. J. 7, 1–22.

[B2] Benjamini Y. Hochberg Y. (1995). Controlling the false discovery rate: A practical and powerful approach to multiple testing. J. R. Stat. Soc. Ser. B 57, 289–300. 10.1111/j.2517-6161.1995.tb02031.x

[B3] Bruijn S. M. Van Impe A. Duysens J. Swinnen S. P. (2012). Split-belt walking: Adaptation differences between young and older adults. J. Neurophysiology 108 (4), 1149–1157. 10.1152/jn.00018.2012 22623488 PMC3424083

[B4] Buurke T. J. W. Lamoth C. J. C. van der Woude L. H. V. den Otter R. (2019). Handrail holding during treadmill walking reduces locomotor learning in able-bodied persons. IEEE Trans. Neural Syst. Rehabilitation Eng. 27 (9), 1753–1759. 10.1109/TNSRE.2019.2935242 31425041

[B5] Buurke T. J. W. Lamoth C. J. C. Vervoort D. van der Woude L. H. V. den Otter R. (2018). Adaptive control of dynamic balance in human gait on a split-belt treadmill. J. Exp. Biol. 221 (13), jeb174896. 10.1242/jeb.174896 29773683

[B6] Caetano M. J. D. Lord S. R. Brodie M. A. Schoene D. Pelicioni P. H. S. Sturnieks D. L. (2018). Executive functioning, concern about falling and quadriceps strength mediate the relationship between impaired gait adaptability and fall risk in older people. Gait Posture 59, 188–192. 10.1016/j.gaitpost.2017.10.017 29055270

[B7] Callisaya M. L. Blizzard L. Schmidt M. D. McGinley J. L. Lord S. R. Srikanth V. K. (2009). A population-based study of sensorimotor factors affecting gait in older people. Age Ageing 38 (3), 290–295. 10.1093/ageing/afp017 19264860

[B8] Charlier R. Mertens E. Lefevre J. Thomis M. (2015). Muscle mass and muscle function over the adult life span: A cross-sectional study in flemish adults. Archives Gerontology Geriatrics 61 (2), 161–167. 10.1016/j.archger.2015.06.009 26164372

[B9] Ekvall Hansson E. Valkonen E. Olsson Möller U. Chen Lin Y. Magnusson M. Fransson P. A. (2021). Gait flexibility among older persons significantly more impaired in fallers than non-fallers-A longitudinal study. Int. J. Environ. Res. Public Health 18 (13), 7074. 10.3390/ijerph18137074 34281011 PMC8297078

[B10] Finley J. M. Bastian A. J. Gottschall J. S. (2013). Learning to be economical: The energy cost of walking tracks motor adaptation. J. Physiology 591 (4), 1081–1095. 10.1113/jphysiol.2012.245506 PMC3591716 23247109

[B11] Hof A. L. (1996). Scaling gait data to body size. Gait Posture 4, 222–223. 10.1016/0966-6362(95)01057-2 11323225

[B12] Krakauer J. W. Hadjiosif A. M. Xu J. Wong A. L. Haith A. M. (2019). Motor learning. Compr. Physiol. 9 (2), 613–663. 10.1002/cphy.c170043 30873583

[B13] Malone L. A. Bastian A. J. (2016). Age-related forgetting in locomotor adaptation. Neurobiol. Learn. Mem. 128, 1–6. 10.1016/j.nlm.2015.11.003 26589520 PMC4839585

[B14] Malone L. A. Bastian A. J. (2010). Thinking about walking: Effects of conscious correction versus distraction on locomotor adaptation. J. Neurophysiology 103 (4), 1954–1962. 10.1152/jn.00832.2009 20147417 PMC2853281

[B15] McAvinue L. P. Habekost T. Johnson K. A. Kyllingsbæk S. Vangkilde S. Bundesen C. (2012). Sustained attention, attentional selectivity, and attentional capacity across the lifespan. Atten. Percept. Psychophys. 74 (8), 1570–1582. 10.3758/s13414-012-0352-6 22825931

[B16] Piasecki M. Ireland A. Jones D. A. McPhee J. S. (2016). Age-dependent motor unit remodelling in human limb muscles. Biogerontology 17 (3), 485–496. 10.1007/s10522-015-9627-3 26667009 PMC4889636

[B17] Ranganathan R. Lee M. H. Newell K. M. (2020). Repetition without repetition: Challenges in understanding behavioral flexibility in motor skill. Front. Psychol. 11, 2018. 10.3389/fpsyg.2020.02018 32903689 PMC7438768

[B18] Reisman D. S. Block H. J. Bastian A. J. (2005). Interlimb coordination during locomotion: What can be adapted and stored? J. Neurophysiology 94 (4), 2403–2415. 10.1152/jn.00089.2005 15958603

[B19] Roemmich R. T. Nocera J. R. Stegemöller E. L. Hassan A. Okun M. S. Hass C. J. (2014). Locomotor adaptation and locomotor adaptive learning in Parkinson's disease and normal aging. Clin. Neurophysiol. 125 (2), 313–319. 10.1016/j.clinph.2013.07.003 23916406 PMC3844121

[B20] Selgrade B. P. Thajchayapong M. Lee G. E. Toney M. E. Chang Y. H. (2017). Changes in mechanical work during neural adaptation to asymmetric locomotion. J. Exp. Biol. 220 (16), 2993–3000. 10.1242/jeb.149450 28596214 PMC5576064

[B21] Sombric C. J. Harker H. M. Sparto P. J. Torres-Oviedo G. (2017). Explicit action switching interferes with the context-specificity of motor memories in older adults. Front. Aging Neurosci. 9, 40. 10.3389/fnagi.2017.00040 28321188 PMC5337495

[B22] Sombric C. J. Torres-Oviedo G. (2020). Augmenting propulsion demands during split-belt walking increases locomotor adaptation of asymmetric step lengths. J. Neuroengineering Rehabilitation 17 (1), 69–y. 10.1186/s12984-020-00698-y PMC7268294 32493440

[B23] Swart S. B. den Otter A. R. Lamoth C. J. C. (2022). Singular spectrum analysis as a data-driven approach to the analysis of motor adaptation time series. Biomed. Signal Process. Control 71, 103068. 10.1016/j.bspc.2021.103068

[B24] Torres-Oviedo G. Bastian A. J. (2012). Natural error patterns enable transfer of motor learning to novel contexts. J. Neurophysiology 107 (1), 346–356. 10.1152/jn.00570.2011 21957223 PMC3349698

[B25] Torres-Oviedo G. Vasudevan E. Malone L. Bastian A. J. (2011). Locomotor adaptation. Prog. Brain Res. 191, 65–74. 10.1016/B978-0-444-53752-2.00013-8 21741544 PMC3738197

[B26] Tyson C. McAndrew C. Tuma P. L. Pegg I. Sarkar A. (2015). Automated nonparametric method for detection of step-like features in biological data sets. Cytometry.Part A J. Int. Soc. Anal. Cytol. 87 (5), 393–404. 10.1002/cyto.a.22631 25652364

[B27] Vandevoorde K. Orban de Xivry J. J. (2019). Internal model recalibration does not deteriorate with age while motor adaptation does. Neurobiol. Aging 80, 138–153. 10.1016/j.neurobiolaging.2019.03.020 31170534

[B28] Vervoort D. Buurke T. J. W. Vuillerme N. HortobÁgyi T. Den Otter R. Lamoth C. J. C. (2020). Adaptive control of dynamic balance across the adult lifespan. Med. Sci. Sports Exerc. 52 (10), 2270–2277. 10.1249/MSS.0000000000002349 32301854 PMC7497469

[B29] Vervoort D. den Otter A. R. Buurke T. J. W. Vuillerme N. Hortobágyi T. Lamoth C. J. C. (2019). Effects of aging and task prioritization on split-belt gait adaptation. Front. Aging Neurosci. 11, 10. 10.3389/fnagi.2019.00010 30760998 PMC6361861

[B30] Winter D. A. Patla A. E. Frank J. S. Walt S. E. (1990). Biomechanical walking pattern changes in the fit and healthy elderly. Phys. Ther. 70 (6), 340–347. 10.1093/ptj/70.6.340 2345777

[B31] Wood J. M. Kim H. E. French M. A. Reisman D. S. Morton S. M. (2020). Use-dependent plasticity explains aftereffects in visually guided locomotor learning of a novel step length asymmetry. J. Neurophysiology 124 (1), 32–39. 10.1152/jn.00083.2020 32432516 PMC7474450

[B32] World Medical Association (2013). World medical association declaration of helsinki: Ethical principles for medical research involving human subjects. Jama 310 (20), 2191–2194. 24141714 10.1001/jama.2013.281053

[B33] Wu G. Cavanagh P. R. (1995). ISB recommendations for standardization in the reporting of kinematic data. J. Biomechanics 28 (10), 1257–1261. 10.1016/0021-9290(95)00017-c 8550644

[B34] Yokoyama H. Sato K. Ogawa T. Yamamoto S. I. Nakazawa K. Kawashima N. (2018). Characteristics of the gait adaptation process due to split-belt treadmill walking under a wide range of right-left speed ratios in humans. PloS One 13 (4), e0194875. 10.1371/journal.pone.0194875 29694404 PMC5918641

